# Supplementary figures and images for: Genomic characterization of novel bat kobuviruses in Madagascar: Implications for viral evolution and zoonotic risk
Source: PLoS One. 2025 Sep 10;20(9):e0331736. doi: 10.1371/journal.pone.0331736 (PMC12422513; doi:10.1371/journal.pone.0331736)

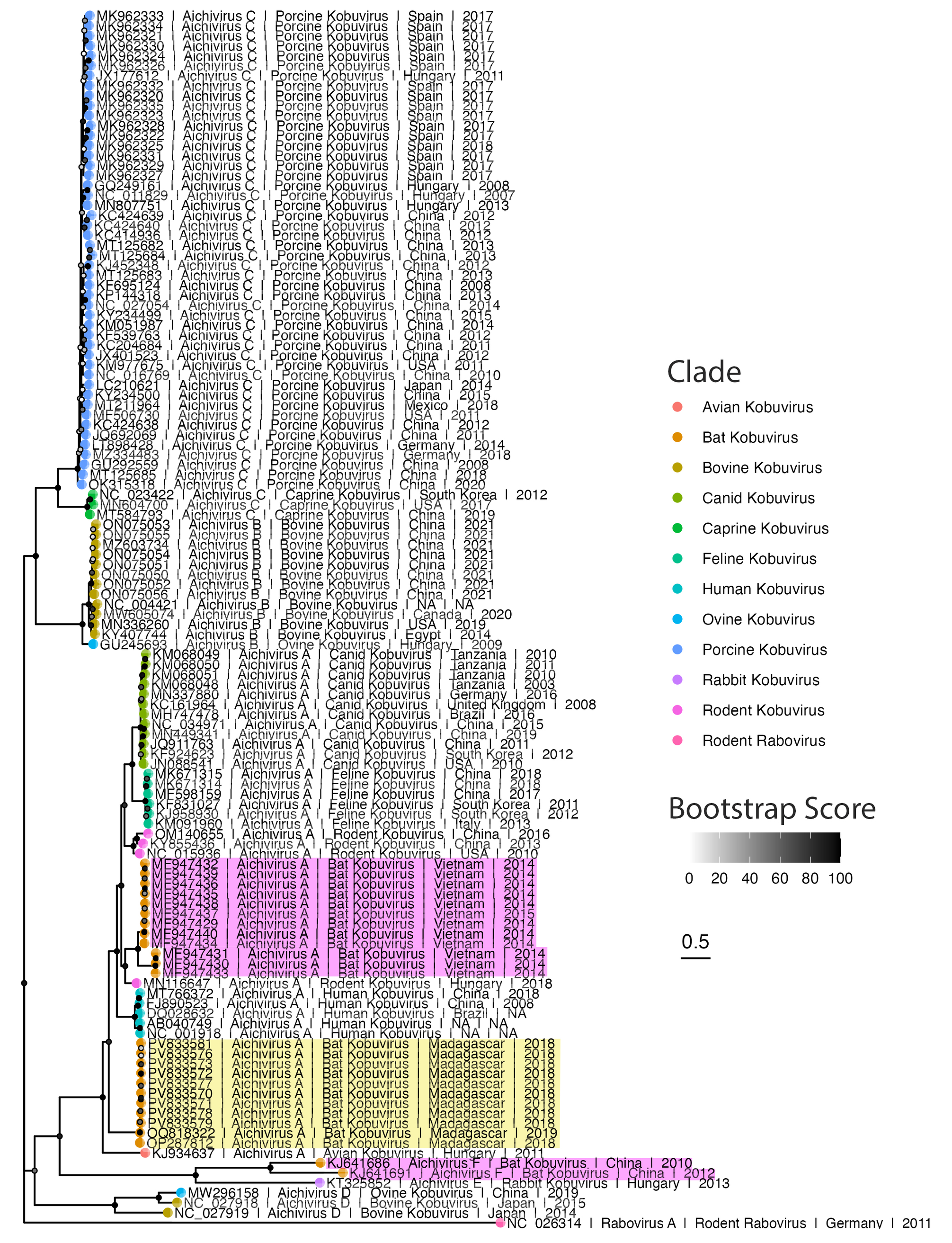

Supplement: S1 Fig — Maximum likelihood phylogeny of kobuvirus sequences (nucleotide substitution model: GTR + I + G4). Node color, represented in greyscale, indicates bootstrap support, with darker shades corresponding to higher support values and lighter shades to lower support values. Madagascar bat kobuvirus sequences are highlighted in yellow, while other bat kobuviruses are highlighted in pink. Tip points are colored by kobuvirus clade. Tip labels include NCBI accession number, virus species, clade, geographic origin, and year of identification, as available from NCBI. Branch lengths are scaled by nucleotide substitution per site, noted by the scalebar. The tree is rooted with rodent rabovirus (NC_026314). The branch length of this outgroup was shortened to improve phylogenetic tree visualization and is denoted as such with a double hash. (TIF) [file pone.0331736.s001.tif]
