# Supplementary material for: Genomic characterization of novel bat kobuviruses in Madagascar: Implications for viral evolution and zoonotic risk
Source: PLoS One. 2025 Sep 10;20(9):e0331736. doi: 10.1371/journal.pone.0331736 (PMC12422513; doi:10.1371/journal.pone.0331736)
Supplement: S1 Table — Summary of minimum information about uncultivated virus genomes (MIUViG) per CheckV and corresponding metadata for all contigs identified as positive kobuvirus hits. Age statuses: NL = Non-Lactating Female, J = Juvenile, A = Adult, P = Pregnant. Accession OQ818322 is marked with an asterisk (*) to note that is derived from urine. (DOCX) [file pone.0331736.s002.docx]

| **ID** | **Length (bp)** | **CZID Alignment Length (bp)** | **CZID**  **Average Read Depth** | **Miuvig Quality** | **Collection Date** | **Location** | **Sex** | **Age Status** |
| --- | --- | --- | --- | --- | --- | --- | --- | --- |
| OQ818322* | 8379 | 1120 | 352X | High-Quality | 23APR2019 | Angavokely | Female | NL |
| OP287812 | 8263 | 6910 | 163X | High-Quality | 27JUL2018 | Angavokely | Female | J |
| PV833573 | 8256 | 6669 | 16X | High-Quality | 09APR2018 | Angavobe | Female | NL |
| PV833572 | 8256 | 6671 | 223X | High-Quality | 09APR2018 | Angavobe | Female | NL |
| PV833579 | 8245 | 6679 | 14X | High-Quality | 15FEB2018 | Angavokely | Male | A |
| PV833571 | 8222 | 6683 | 76X | High-Quality | 09SEP2018 | Angavobe | Female | P |
| PV833581 | 8214 | 6651 | 241X | High-Quality | 09APR2018 | Angavobe | Female | NL |
| PV833578 | 8212 | 6678 | 10X | High-Quality | 15FEB2018 | Angavokely | Male | J |
| PV833577 | 8201 | 6669 | 9X | High-Quality | 09APR2018 | Angavobe | Male | A |
| PV833576 | 8195 | 6651 | 46X | High-Quality | 09APR2018 | Angavobe | Female | NL |
| PV833570 | 8190 | 6651 | 24X | High-Quality | 27JUL2018 | Angavokely | Male | J |
| PV833582 | 3651 | 2839 | 5X | Genome-Fragment | 09SEP2018 | Angavobe | Female | P |
| OR082796 | 2077 | 1120 | 1852X | Genome-Fragment | 09APR2018 | Angavobe | Female | NL |
| PV833574 | 1536 | 1458 | 1X | Genome-Fragment | 27JUL2018 | Angavokely | Male | A |
| PV833580 | 1358 | 1120 | 4X | Genome-Fragment | 09SEP2018 | Angavobe | Male | A |
| PV833575 | 1304 | 1288 | 5X | Genome-Fragment | 09APR2018 | Angavobe | Female | NL |
