# Supplementary material for: Genomic characterization of novel bat kobuviruses in Madagascar: Implications for viral evolution and zoonotic risk
Source: PLoS One. 2025 Sep 10;20(9):e0331736. doi: 10.1371/journal.pone.0331736 (PMC12422513; doi:10.1371/journal.pone.0331736)
Supplement: S2 Table — Summary of reference sequences used to annotate the OP287812 genome and their corresponding NCBI accession number, including the percent similarity between the reference sequence and OP287812. Columns without data indicate regions that were manually annotated (i.e., 5’ UTR, L Peptide, 3’ UTR) without the use of a reference sequence. (DOCX) [file pone.0331736.s003.docx]

| **Genome Region** | **Start (nt)** | **End (nt)** | **% Similarity To NCBI Reference** | **NCBI Accession** |
| --- | --- | --- | --- | --- |
| 5’UTR | 1 | 679 | - | - |
| ORF | 680 | 7984 | 71.37% | JN387133 |
| L | 680 | 1234 | - | - |
| VP0 | 1235 | 2347 | 71.17% | FJ890523 |
| VP3 | 2348 | 3016 | 78.42% | FJ890523 |
| VP1 | 3017 | 3742 | 67.72% | JQ898342 |
| 2A | 3743 | 4150 | 70.10% | MH747478 |
| 2B | 4151 | 4645 | 75.35% | JQ898342 |
| 2C | 4646 | 5650 | 77.41% | FJ890523 |
| 3A | 5651 | 5929 | 65.37% | MH747478 |
| 3B | 5930 | 6007 | 67.07% | JN387133 |
| 3C | 6008 | 6577 | 75.74% | MH747478 |
| 3D | 6578 | 7984 | 80.98% | JN387133 |
| 3’UTR | 7985 | 8263 | - | - |
