# Supplementary material for: Genomic characterization of novel bat kobuviruses in Madagascar: Implications for viral evolution and zoonotic risk
Source: PLoS One. 2025 Sep 10;20(9):e0331736. doi: 10.1371/journal.pone.0331736 (PMC12422513; doi:10.1371/journal.pone.0331736)
Supplement: S3 Table — Identity percentages, E-Values, corresponding hit lengths, and NCBI Accessions for the highest-ranking BLASTx hit between OP287812 and NCBI kobuvirus sequences. Amino acid lengths for the OP287812 genome, as well as predicted protease cleavage sites (in single-letter amino acid code) indicating start and end positions for individual proteins, are listed. No hits were observed within the 5’UTR and 3’UTR. (DOCX) [file pone.0331736.s004.docx]

| **Genome Region** | **Start (aa)** | **End (aa)** | **Predicted Protease Cleavage Site** | **Pairwise Identity %** | **E-Value** | **NCBI Accession** | **Hit Length (bp)** |
| --- | --- | --- | --- | --- | --- | --- | --- |
| Whole Genome | 1 | 2754 | - | 78.10% | 0 | AWK02691 | 2409 |
| ORF | 227 | 2661 | - | 78.10% | 0 | AWK02691 | 2409 |
| L | 227 | 411 | Q/G | 98.90% | 5.38E-128 | AGL97808 | 184 |
| VP0 | 412 | 782 | Q/H | 82.00% | 3.89E-164 | QUJ18037 | 372 |
| VP3 | 783 | 1005 | Q/A | 87.50% | 2.65E-119 | AVH76467 | 223 |
| VP1 | 1006 | 1247 | Q/G | 77.60% | 1.65E-106 | AVH76458 | 222 |
| 2A | 1248 | 1383 | Q/G | 74.30% | 6.06E-42 | QIE07158 | 135 |
| 2B | 1384 | 1548 | Q/G | 84.80% | 6.22E-87 | AVH76472 | 164 |
| 2C | 1549 | 1883 | Q/G | 86.90% | 0 | AFV70595 | 334 |
| 3A | 1884 | 1976 | Q/A | 64.90% | 1.23E-31 | QUJ18037 | 92 |
| 3B | 1977 | 2002 | Q/G | 88.90% | 1.53E-04 | AXE75324 | 25 |
| 3C | 2003 | 2192 | Q/S | 78.90% | 3.26E-101 | QEV86991 | 189 |
| 3D | 2193 | 2661 | - | 88.50% | 0 | YP_004782207 | 467 |
