# Supplementary material for: Genomic characterization of novel bat kobuviruses in Madagascar: Implications for viral evolution and zoonotic risk
Source: PLoS One. 2025 Sep 10;20(9):e0331736. doi: 10.1371/journal.pone.0331736 (PMC12422513; doi:10.1371/journal.pone.0331736)
Supplement: S4 Table — Alignment statistics between bat kobuvirus sequences used in Supplemental Figure 1. Accession OQ818322 is marked with an asterisk (*) to note that is derived from urine. (DOCX) [file pone.0331736.s005.docx]

| **Sequence ID** | **Country** | **Host** | **Percent Identity (%)** | **Percent Coverage (%)** | **Mismatches** | **Length (bp)** |
| --- | --- | --- | --- | --- | --- | --- |
| OR082796 | Madagascar | *E. dupreanum* | 97.29 | 24.53 | 55 | 2077 |
| OQ818322* | Madagascar | *E. dupreanum* | 98.46 | 100 | 76 | 8379 |
| PV833573 | Madagascar | *E. dupreanum* | 97.91 | 99.32 | 158 | 8256 |
| PV833572 | Madagascar | *E. dupreanum* | 98.03 | 99.32 | 162 | 8256 |
| PV833579 | Madagascar | *E. dupreanum* | 98.43 | 99.32 | 129 | 8245 |
| PV833571 | Madagascar | *E. dupreanum* | 98.03 | 99.32 | 162 | 8222 |
| PV833581 | Madagascar | *E. dupreanum* | 98.11 | 99.24 | 155 | 8214 |
| PV833578 | Madagascar | *E. dupreanum* | 98.16 | 99.26 | 151 | 8212 |
| PV833577 | Madagascar | *E. dupreanum* | 98.12 | 99.24 | 154 | 8201 |
| PV833576 | Madagascar | *E. dupreanum* | 98.11 | 99.18 | 155 | 8195 |
| PV833570 | Madagascar | *E. dupreanum* | 98.11 | 99.12 | 155 | 8190 |
| PV833582 | Madagascar | *E. dupreanum* | 99.97 | 44.18 | 1 | 3651 |
| PV833574 | Madagascar | *E. dupreanum* | 99.87 | 18.59 | 2 | 1536 |
| PV833580 | Madagascar | *E. dupreanum* | 99.85 | 16.43 | 2 | 1358 |
| PV833575 | Madagascar | *E. dupreanum* | 98.70 | 15.78 | 17 | 1304 |
| MF947440 | Vietnam | *S. kuhlii* | 71.94 | 76.21 | 1726 | 6354 |
| MF947439 | Vietnam | *S. kuhlii* | 71.84 | 76.21 | 1732 | 6354 |
| MF947438 | Vietnam | *S. kuhlii* | 72.02 | 82.16 | 1845 | 6859 |
| MF947437 | Vietnam | *S. kuhlii* | 72.02 | 82.16 | 1845 | 6859 |
| MF947436 | Vietnam | *S. kuhlii* | 71.56 | 71.09 | 1630 | 5931 |
| MF947435 | Vietnam | *S. kuhlii* | 71.83 | 75.70 | 1721 | 6312 |
| MF947434 | Vietnam | *S. kuhlii* | 71.96 | 75.70 | 1713 | 6312 |
| MF947433 | Vietnam | *S. kuhlii* | 69.09 | 64.83 | 1631 | 5393 |
| MF947432 | Vietnam | *S. kuhlii* | 71.80 | 76.16 | 1734 | 6350 |
| MF947431 | Vietnam | *S. kuhlii* | 69.70 | 68.15 | 1681 | 5667 |
| MF947430 | Vietnam | *S. kuhlii* | 69.80 | 67.57 | 1661 | 5619 |
| MF947429 | Vietnam | *S. kuhlii* | 72.05 | 76.04 | 1715 | 6340 |
| KJ641691 | China | *M. fuliginosus* | 45.70 | 83.61 | 3258 | 7279 |
| KJ641686 | China | *M. ricketti* | 47.77 | 87.35 | 3169 | 7555 |
