# Supplementary material for: Genomic characterization of novel bat kobuviruses in Madagascar: Implications for viral evolution and zoonotic risk
Source: PLoS One. 2025 Sep 10;20(9):e0331736. doi: 10.1371/journal.pone.0331736 (PMC12422513; doi:10.1371/journal.pone.0331736)
Supplement: Attachment — Submitted filename: Response to Reviewers.docx [file pone.0331736.s008.docx]

Dear reviewers,

Thank you for giving us the opportunity to submit a revised draft of our manuscript. We appreciate the time and effort that you have dedicated to providing your valuable feedback on our manuscript. We are grateful for the insightful comments and find the manuscript much improved as a result of your suggestions. We have been able to incorporate your suggested changes, which are now reflected within the manuscript.

**Rebuttal:**

***Journal Requirements –***

- Ensure that manuscript meets journal’s style requirements, including those for file names and section headers.

**Response:**

- We added the email to the corresponding author on the title page of the manuscript and supplementary file.
- We corrected the section headers to follow the journal guidelines (Header 1, Bold, 18pt font) within the manuscript.
- We corrected the sub-section headers to follow journal guidelines (Header 2, Bold, 16pt font) within the manuscript.
- We added a section for the supporting information after the references on the main manuscript.

***Journal Requirements Continued –***

- Review reference list to make sure it is complete and correct.
  - Check that none of them are retracted, and either replace retracted articles or give a rationale for why they are being cited.
  - Changes to the reference list should be mentioned in the rebuttal letter that accompanies revised manuscript.

**General changes to citations are listed below:**

- We checked each citation to confirm that none of them were retracted.
- We added a citation, now citation 9. This citation was necessary to add, as it is the first published piece of literature on kobuviruses and serves as a good paper for the introduction of this viral clade. The citation is listed below:
  - [9] Yamashita T, Kobayashi S, Sakac K, Nakata S, Chiba S, Ishihara Y, et al. Isolation of Cytopathic Small Round Viruses with BS-C-l Cells from Patients with Gastroenteritis. J Infect Dis 1991;164:954–7. https://doi.org/10.1093/infdis/164.5.954.
- We flipped the order of citation 13 and citation 14 to better reflect the order in which we present animal hosts of Aichivirus B-F genotypes and their corresponding papers. The new order is listed below:
  - [13] Mohamed FF, Mansour SMG, Orabi A, El-Araby IE, Ng TFF, Mor SK, et al. Detection and genetic characterization of bovine kobuvirus from calves in Egypt. Arch Virol 2018;163:1439–47. https://doi.org/10.1007/s00705-018-3758-1.
  - [14] Wang L, Fredrickson R, Duncan M, Samuelson J, Hsiao S-H. Bovine Kobuvirus in Calves with Diarrhea, United States - Volume 26, Number 1—January 2020 - Emerging Infectious Diseases journal - CDC n.d. https://doi.org/10.3201/eid2601.191227.
- We retained citation 18, removed citation 50, and repositioned citation 51. Citation 18 and 51 are now numbered differently on the updated reference list, respectively listed below:
  - [12] Lu L, Van Dung N, Ivens A, Bogaardt C, O’Toole A, Bryant JE, et al. Genetic diversity and cross-species transmission of kobuviruses in Vietnam. Virus Evol 2018;4:vey002. https://doi.org/10.1093/ve/vey002.
  - [63] Cui Y, Li J, Guo J, Pan Y, Tong X, Liu C, et al. Evolutionary Origin, Genetic Recombination, and Phylogeography of Porcine Kobuvirus. Viruses 2023;15:240. https://doi.org/10.3390/v15010240.
- We moved what was originally citation 52 to position 62 within our bibliography. We cited the BEAST software twice before, now citing it once when it is originally mentioned. Now, both BEAST and BEAST2 are cited side-by-side when originally mentioning the software. The new order is listed below:
  - [58] Bouckaert R, Vaughan TG, Barido-Sottani J, Duchêne S, Fourment M, Gavryushkina A, et al. BEAST 2.5: An advanced software platform for Bayesian evolutionary analysis. PLoS Comput Biol 2019;15:e1006650. https://doi.org/10.1371/journal.pcbi.1006650.
  - [59] Drummond AJ, Rambaut A. BEAST: Bayesian evolutionary analysis by sampling trees. BMC Evol Biol 2007;7:214. https://doi.org/10.1186/1471-2148-7-214.
- We combined what were originally the last two paragraphs of the discussion, which spoke about the lack in understanding of the geographical distribution of kobuvirus and the use of existing tools to expand upon that understanding (lines 767-794 in ‘Manuscript With Track Changes’ & lines 448-466 in ‘Manuscript’). We combined these to make room for the addition of two later sections that Reviewer 1 had requested, which we reference later in the document. We also combined parts of the second and third paragraph of the discussion to make room for these later additions to the discussion (lines 624-648 in ‘Manuscript With Track Changes’ & lines 409-427 in ‘Manuscript’). This meant that we had to change the order of a few citations, or add some new ones. Those changes are listed below:
  - [63] Cui Y, Li J, Guo J, Pan Y, Tong X, Liu C, et al. Evolutionary Origin, Genetic Recombination, and Phylogeography of Porcine Kobuvirus. Viruses 2023;15:240. https://doi.org/10.3390/v15010240.
  - [65] Khamrin P, Maneekarn N, Hidaka S, Kishikawa S, Ushijima K, Okitsu S, et al. Molecular detection of kobuvirus sequences in stool samples collected from healthy pigs in Japan. Infect Genet Evol J Mol Epidemiol Evol Genet Infect Dis 2010;10:950–4. <https://doi.org/10.1016/j.meegid.2010.06.001>.
  - [73] Han X, Zhang W, Xue Y, Shao S. Sequence analysis reveals mosaic genome of Aichi virus. Virol J 2011;8:390. <https://doi.org/10.1186/1743-422X-8-390>.
  - [75] Lescar J, Canard B. RNA-dependent RNA polymerases from flaviviruses and Picornaviridae. Curr Opin Struct Biol 2009;19:759–67. https://doi.org/10.1016/j.sbi.2009.10.011.
  - [77] Reuter G, Boldizsár Á, Pankovics P. Complete nucleotide and amino acid sequences and genetic organization of porcine kobuvirus, a member of a new species in the genus Kobuvirus, family Picornaviridae. Arch Virol 2009;154:101–8. https://doi.org/10.1007/s00705-008-0288-2.
- We removed citation 61. Though it speaks to the anthropogenic effects on bat populations through the introduction of domestic cats into wild habitats, it does not specifically reference Madagascar, which is what the passage it was referenced in was doing. That citation is listed below:
  - [61] Rocha R. Look what the cat dragged in: Felis silvestris catus as predators of insular bats and instance of predation on the endangered Pipistrellus maderensis. Barbastella 2015;8
- The citations below were included in an original copy of the working manuscript prior to our initial submission, but we failed to notice that the citation software we used did not remove them from the bibliography even though they were not cited in-text. They are now removed from the updated document, and are listed below:
  - [62] (Turner PE, Elena SF. Cost of Host Radiation in an RNA Virus. Genetics 2000;156:1465–70. <https://doi.org/10.1093/genetics/156.4.1465>)
  - [70] (Lau SKP, Woo PCY, Yip CCY, Li KSM, Fan RYY, Bai R, et al. Chickens host diverse picornaviruses originated from potential interspecies transmission with recombination. J Gen Virol 2014;95:1929–44. <https://doi.org/10.1099/vir.0.066597-0>),
  - [71] (Li H, Mendelsohn E, Zong C, Zhang W, Hagan E, Wang N, et al. Human-animal interactions and bat coronavirus spillover potential among rural residents in Southern China. Biosaf Health 2019;1:84–90. https://doi.org/10.1016/j.bsheal.2019.10.004).

***Journal Requirements Continued –***

- State what role the funders took in this study. If funders had no role, state “The funders had no role in study design, data collection and analysis, decision to publish, or preparation of the manuscript.”
  - If this is incorrect, amend as needed.
  - *Include the amended Role of Funder statement in cover letter; PLOS will change submission form on our behalf.*

**Response:**

- We stated that the funders had no role in design, data collection and analysis, decision to publish, or preparation of the manuscript at the end of the funding statement.

***Reviewer comments to author -***

- Recommend strengthening the manuscript further, particularly the broader implications of the findings for zoonotic risk.
  a. In the introduction, it would be valuable to discuss the specific ecological or biological factors that make kobuviruses in bats (significant) even if other wildlife species are more prone to cross-species transmission.
  b. Discuss the specific mechanisms of cross-species transmission of kobuviruses between different wildlife species.
  c. Include more information about the sequencing depth and any steps taken to ensure data quality in regards to mNGS sequencing.

**Response:**

- *In regards to comment a)*
  a. We added a paragraph in the intro speaking to what is particularly interesting about bat kobuviruses. This paragraph speaks to bat characteristics that make them suitable hosts for a wide array of zoonotic and virulent pathogens. This paragraph includes some comment about how these traits, in combination with what is known about kobuviruses in other hosts, might contribute to bat kobuvirus distribution (lines 93-109 ‘Manuscript With Track Changes’ & lines 77-93 in ‘Manuscript’). The inclusion of this paragraph resulted in the addition of 9 new citations. These are listed as citations #22-30 in the manuscript bibliography. Their addition altered the original numbering of all citations which came after.
- *In regards to comment b)*
  - We included a new paragraph on the mechanisms that may drive cross-species transmission of kobuviruses between differing species. This is now paragraph three of the discussion (lines 651-658, 757-765 in ‘Manuscript With Track Changes’ & lines 430-446 in ‘Manuscript’), which speaks to the understood mechanism of transmission (fecal-oral) between hosts, as well as more mechanistic propositions for what may be driving kobuvirus transmission between hosts. The inclusion of this paragraph resulted in the addition of 8 new citations. These are listed as citations #66-73 in the manuscript bibliography. Their addition altered the original numbering of all citations which came after.
- *In regards to comment c)*
  - We added a description regarding the sequencing depth and quality filtering corresponding to our project (lines 292-299 in ‘Manuscript With Track Changes’ & lines 184-191 in ‘Manuscript’) and similar steps taken for our near-full representative genome (lines 347-350 in ‘Manuscript With Track Changes’ & lines 232-234 in ‘Manuscript’). Upon re-evaluating our analysis, we also identified an error in the original coverage figure for OP287812: the y-axis was labeled as reads per million, but we displayed the raw read counts. We have now corrected the figure to accurately reflect reads per million by scaling the raw read counts accordingly.

***Editor comments -***

- Authors should mention in the introduction or discussion if there are social, cultural, or anthropogenic activities peculiar to Madagascar that encourages human-bat contact and constitute public health risks.

**Response:**

- We added a concluding paragraph at the end of the discussion speaking to the social, cultural, and anthropogenic activities that are unique to Madagascar, which contribute to the human-bat contacts in the country (lines 797-809 in ‘Manuscript With Track Changes’ & lines 469-481 in ‘Manuscript’). We speak about the anthropogenic effects that lead to downstream synanthropic lifestyles by bats, and future directions for both conservationists and disease ecologists on the island. The inclusion of this paragraph resulted in the addition of 13 new citations. These are listed as citations #77-89 in the manuscript bibliography. Their addition altered the original numbering of all citations which came after.

**Other changes to manuscript:**

- Addition of new kobuvirus genomes
  - During re-analysis of kobuvirus-positive hits in CZID, we identified 13 contigs (>1,000bp) that were assigned to the kobuvirus genus by CZID but were not captured in our offline BLAST validation step. Although each of these samples only contained a single kobuvirus-assigned contig, the contigs were sufficiently long and identified with high confidence by CZID. They also demonstrated >98% genome identity to our original kobuvirus genome, OP287812. We opted to include them and have clarified this exception in the manuscript. We also now report another kobuvirus-aligned contig identified in the mNGS data from a urine sample. This contig exhibited strong genomic and phylogenetic similarity to the fecal-derived sequences, so we deemed this likely contamination from feces. We address this within line 404 of the manuscript.
  - Due to this change, we have made changes to Table 1 and its corresponding text (lines 402-482 in ‘Manuscript With Track Changes’ & lines 267-293 in ‘Manuscript’), which now reflects the updated count of kobuvirus-positive samples. We also added a new supplementary table to the supplemental text (Supplemental Table 1), which gives some metadata and results from our minimum information about uncultivated virus genomes (MIUViG) search per CheckV. This shifted the order of the supplementary information that followed. These results can also be found on our updated GitHub. We also aligned our newly identified contigs to our representative genome OP287812 and report summary statistics in our genome alignment table within the supplement (Supplemental Table 4). Lastly, we created a new maximum-likelihood phylogenetic tree with the larger contigs we identified (>8,000bp) (Supplementary Figure 1), in addition to the previous sequences included in this analysis. We found that our newly added sequences nest within a monophyletic clade, including OP287812, so we opted to deem OP287812 as the representative sequence for the rest of the analyses throughout the paper, which we kept the same throughout.
